# Effectiveness of the Care of Persons With Dementia in Their Environments Intervention When Embedded in a Publicly Funded Home- and Community-Based Service Program

**DOI:** 10.1093/geroni/igaa053

**Published:** 2020-10-26

**Authors:** Richard H Fortinsky, Laura N Gitlin, Laura T Pizzi, Catherine Verrier Piersol, James Grady, Julie T Robison, Sheila Molony, Dorothy Wakefield

**Affiliations:** 1 Center on Aging, School of Medicine, University of Connecticut, Farmington, USA; 2 College of Nursing and Health Professions, Drexel University, Philadelphia, Pennsylvania, USA; 3 Center for Health Outcomes Research, Rutgers University, Piscataway, New Jersey, USA; 4 College of Health Professions, Thomas Jefferson University, Philadelphia, Pennsylvania, USA; 5 Department of Public Health Sciences, School of Medicine, University of Connecticut, Farmington, USA; 6 School of Nursing, Quinnipiac University, North Haven, Connecticut, USA

**Keywords:** Family caregivers, Medicaid waiver programs, Pragmatic trial elements

## Abstract

**Background and Objectives:**

In the absence of effective pharmacotherapy, there is an urgent need to test evidence-based dementia care interventions using pragmatic trial approaches. We present results from a study in which an evidence-based, nonpharmacologic intervention for persons living with Alzheimer’s disease and related dementia (ADRD) and their informal caregivers, Care of Persons with Dementia in their Environments (COPE), was tested in a Medicaid and state revenue-funded home and community-based service (HCBS) program.

**Research Design and Methods:**

Using pragmatic trial design strategies, persons living with ADRD and their caregivers were randomly assigned as dyads to receive COPE plus usual HCBS (COPE; *n* = 145 dyads) or usual HCBS only (Usual Care or UC; *n* = 146 dyads). Outcomes were measured prerandomization, and 4 and 12 months postrandomization. Outcomes for persons living with ADRD included functional independence, activity engagement, self-reported quality of life, and behavioral and psychological symptoms. Caregiver outcomes included perceived well-being, confidence using dementia management strategies, and degree of distress caused by behavioral and psychological symptoms.

**Results:**

After 4 months, caregivers receiving COPE reported greater perceived well-being (least squares mean = 3.2; 95% CI: 3.1–3.3) than caregivers receiving UC (3.0; 2.9–3.0; *p* < .001), and persons living with ADRD receiving COPE, compared to those receiving UC, showed a strong trend toward experiencing less frequent and less severe behavioral and psychological symptoms (9.7; 5.2–14.2 vs 12.7; 8.3–17.1; *p* = .07). After 12 months, persons living with ADRD receiving COPE were more engaged in meaningful activities (2.1; 2.0–2.1 vs 1.9; 1.9–2.0; *p* = .02) than those receiving UC.

**Discussion and Implications:**

Embedding COPE in a publicly funded HCBS program yielded positive immediate effects on caregivers’ well-being, marginal positive immediate effects on behavioral and psychological symptoms, and long-term effects on meaningful activity engagement among persons living with ADRD. Findings suggest that COPE can be effectively integrated into this service system, an important step towards widespread adoption.

**Clinical Trials Registration Number:**

NCT02365051.

**Translational Significance:** Using a randomized design, this study embedded an evidence-based, in-home dementia care program called *Care of Persons with Dementia in their Environments (COPE)* in a state-level Medicaid and state revenue-funded home and community-based service program. Findings revealed that family caregivers of older adults benefitted from COPE through improved overall well-being due to learned dementia management skills, while older adults living with dementia benefitted chiefly from sustained engagement in meaningful activities. This effectiveness study demonstrates that COPE can yield positive value when delivered in pragmatic fashion to older adults enrolled in a service system that operates in a large majority of states in the United States.

## Background and Objectives

Dementia, an umbrella term encompassing multiple causes of brain neurodegeneration that result in cognitive decline and development of behavioral and psychological symptoms, affected more than 50 million people worldwide in 2019; by 2050, this number will reach more than 150 million people (Alzheimer’s Disease International, 2019). In 2020, an estimated 5.8 million Americans are living with Alzheimer’s disease and related dementia (ADRD), and more than 16 million informal caregivers, mostly family members (hereafter, *caregivers*), provide unpaid care to these individuals (Alzheimer’s Association, 2020).

In the absence of effective pharmacotherapy to treat ADRD or appreciably slow symptom progression, numerous nonpharmacologic interventions designed to help persons living with ADRD and caregivers have been implemented and evaluated. Meta-analyses and scoping reviews of systematic reviews of these interventions have found evidence of efficacious programs, particularly on improving skill-building and psychological outcomes of caregivers of persons living with ADRD (Butler et al., 2020; Chen & Zheng, 2020; Gaugler et al., 2020; Gitlin, Jutkowitz, et al., 2020). Investigators are increasingly turning their attention to replicating or adapting efficacious interventions for persons living with ADRD and their caregivers in “real-world” health care and social service systems and settings. Such efforts have involved translational studies that identify adaptations needed to evidence-based programs in dementia care that are required to embed them in health care systems in a more pragmatic fashion (Fortinsky et al., 2016; Gitlin & Czaja, 2016; Gitlin et al., 2015). Most recently, published translational studies using single group pretest-posttest designs found that beneficial outcomes for persons living with ADRD and/or caregivers could be achieved when efficacious interventions are incorporated into community service settings (Bass et al. 2019; Cho et al., 2019; Hodgson & Gitlin, in press).

In this study containing pragmatic trial elements (Loudon et al., 2015), we incorporated the efficacious Care of Persons with Dementia in their Environments (COPE) intervention (Gitlin et al., 2010) into the Connecticut Home Care Program for Elders (CHCPE), a Medicaid and state revenue-funded home and community-based service program for older adults at high risk for nursing home admission (Connecticut State Department of Social Services, 2018). The COPE intervention features up to 10 in-home visits by an occupational therapist (OT) and a single home visit and follow-up telephone contact by an advanced practice nurse (APN). The COPE intervention is grounded in principles of the competence-environmental press theory, which argues that health and well-being is optimized when the physical and social environment is aligned with individual competencies (Lawton & Nahemow, 1973). COPE seeks to maximize physical function and quality of life in persons living with ADRD and build dementia management skills in caregivers by realigning capabilities of persons living with ADRD with environmental demands, particularly the physical environment of the home setting (Fortinsky et al., 2016; Gitlin et al., 2010). In an efficacy clinical trial, persons living with ADRD receiving COPE experienced less functional decline and more activity engagement, compared to an attention control group. Caregivers receiving COPE, compared to controls, reported improved well-being, increased confidence in using a range of nonpharmacological strategies (e.g., communications, task and environmental simplification) to address dementia symptoms, and greater ability to keep their family member at home (Gitlin et al., 2010).

We chose to incorporate COPE into the CHCPE, for several reasons. First, most states are aggressively rebalancing the long-term services and supports components of their Medicaid programs for cost containment and consumer preference purposes. Increasing proportions of Medicaid beneficiaries regardless of age who require long-term services and supports receive such care in home settings rather than in nursing homes (Kaye, 2012). Second, more than 40 states operate the same type of Medicaid home and community-based service (HCBS) waiver program as the Medicaid component of the CHCPE in Connecticut (Watts et al., 2020). For both reasons, lessons learned about the value of COPE in this study will be of interest to most state Medicaid programs in the country. Third, although 25%–30% of the 16,000 unduplicated CHCPE clients served annually have a diagnosis of Alzheimer’s disease or other dementia, the CHCPE offers services designed to support activities of daily living, rather than evidence-based interventions tailored to address cognitive, environmental, and/or behavioral challenges that could affect health-related outcomes and sustain living at home for these clients. Moreover, no CHCPE services offer skill-building and supportive services to family caregivers of CHCPE clients with dementia to help improve their dementia-related symptom management skills and health-related outcomes.

To our knowledge, the study reported here is the first to employ an embedded pragmatic trial approach and a randomized design to test a proven dementia care program that includes family caregivers and persons living with ADRD enrolled in a publicly funded HCBS program. The in-home nature of the COPE intervention and its focus on optimizing function and independent living is highly consistent with the programmatic goals of Medicaid and state revenue-funded HCBS programs for older adults nationwide. Embedding and testing evidence-based interventions for persons living with ADRD in existing publicly funded home care programs and their caregivers can help inform state and federal governments as to the added value of augmenting existing services and whether doing so can delay or avoid Medicaid-covered nursing home admissions and Medicare-covered hospitalizations.

In this paper, we report on the effectiveness of the COPE intervention by focusing on clinically meaningful dementia-related outcomes for persons living with ADRD and dementia management-related outcomes for their caregivers. For persons living with ADRD, we determine COPE effect on functional independence, engagement in activities, quality of life, and prevention or alleviation of behavioral and psychological symptoms, 4 and 12 months after randomization. We hypothesized that persons living with ADRD receiving COPE plus usual care would show greater functional independence, greater engagement in activities, better quality of life, and less frequent and less severe behavioral and psychological symptoms, compared to those receiving usual care only, 4 and 12 months after randomization. For caregivers, we determine COPE effect on perceived well-being, confidence in using dementia management strategies, and level of distress related to behavioral and psychological symptoms of persons living with ADRD 4 and 12 months after randomization. We hypothesized that caregivers receiving COPE plus usual care will report improvement in all specified outcomes compared to those receiving usual care only, 4 and 12 months after randomization.

## Research Design and Methods

### Study Design

This study was designed conceptually as an effectiveness–implementation hybrid design (Curran et al., 2012). This hybrid design blends components of clinical effectiveness and implementation research, allowing conclusions to be drawn simultaneously about the effectiveness of an evidence-based nonpharmacologic intervention on meaningful health-related outcomes at the individual level in a real-world service setting, and about the degree of success in incorporating the intervention into that service setting from the viewpoints of feasibility, acceptability, sustainability, and cost–benefit (Curran et al., 2012). This paper reports on main outcomes relevant to the effectiveness component of the study design, focusing on both persons living with ADRD and caregivers as individuals under study.

We employed a stratified randomized design in this effectiveness study. Stratification was based on CHCPE eligibility categories that distinguished older adults who were: (a) functionally eligible for nursing home admission and financially eligible for Medicaid (Medicaid-funded); or (b) functionally eligible for nursing home admission but not financially eligible for Medicaid (state revenue-funded). We wanted to ensure that the COPE and usual care (UC) groups had approximately equal proportions of older adults who were Medicaid-funded and state revenue-funded because each category has a different monthly budget cap on the amount of CHCPE services allowed. Therefore, within their stratum, persons living with ADRD and their caregivers were randomly assigned as dyads to receive either: usual CHCPE services (usual care) with no COPE intervention or the COPE intervention plus usual care.

### Target Population and Study Setting

The study’s target population was older adults (age ≥65 years) with dementia who received services from the CHCPE and their informal caregivers. The CHCPE is administered in Connecticut by four care management organizations, each responsible for a distinct geographic area, that employ care managers who plan and coordinate services for the CHCPE client population. For this study, we partnered with Connecticut Community Care, the largest of the four care management organizations in Connecticut. Established in 1979 as a pioneer care management organization for older adults, today Connecticut Community Care provides care management services to more than 8,000 CHCPE clients daily. Connecticut Community Care shares important features of organizations that are responsible for administering their states’ Medicaid- and state revenue-funded HCBS programs for older adults. Shared features include conducting uniform clinical assessments of clients for eligibility determination and care plan development, coordinating a range of direct service providers delivering HCBS, and monitoring the cost of care plans for each client. Accordingly, lessons learned in this study about the effectiveness of COPE will be of interest to organizations responsible for managing Medicaid- and state-funded HCBS programs throughout the United States.

### Eligibility Criteria for Persons Living With ADRD and Caregivers

In keeping with a pragmatic clinical trials approach, study entry criteria were designed to accommodate data available from the Connecticut Community Care electronic database.

*Inclusion criteria for persons living with ADRD*: (a) Active CHCPE client; (b) diagnosis of dementia or four or more errors on the Mental Status Questionnaire, considered moderate cognitive impairment (Kahn et al., 1960); and (c) speaks or understands English.
*Exclusion criteria for persons living with ADRD:* (a) Diagnosed schizophrenia or bipolar disorder; (b) bedbound and unresponsive; (c) participation in concurrent experimental drug study designed to treat agitation; and (d) home environment deemed unsafe and/or unsanitary.
*Caregiver inclusion criteria:* (a) ≥21 years of age; (b) willing and able to participate in all aspects of the study; (c) plans to live in area for 12 months; and (d) speaks English.
*Caregiver exclusion criteria:* (a) Terminal illness with life expectancy of <12 months; (b) participation in concurrent nonpharmacologic trial designed to help caregivers of people living with ADRD; and (c) planning to admit person living with ADRD to a nursing home within 6 months.

### Recruitment, Enrollment, and Randomization

In keeping with our pragmatic trial approach, we embedded recruitment within the daily operations of Connecticut Community Care. During routine monthly clinical monitoring telephone calls, care managers at Connecticut Community Care explained key study features to provisionally eligible clients or their caregivers and referred interested dyads to the research study coordinator. Research staff then conducted telephone screenings with caregivers to determine whether clients and caregivers fulfilled all remaining inclusion and exclusion criteria. Consenting and prerandomization data collection occurred in the home setting. Randomization was stratified according to whether the CHCPE client received financial support from Medicaid funds or from state general revenues. The study protocol was approved by the UConn Health Institutional Review Board.

### Intervention Condition

Details about the COPE intervention as implemented in this study have been published elsewhere (Fortinsky et al., 2016). Briefly, in addition to receiving HCBS from the CHCPE program, dyads randomly assigned to the COPE treatment group received up to 10 in-home visits by OTs, and one in-home visit and one follow-up telephone call by an APN. Per COPE intervention protocol, during the first two home visits, OTs interviewed caregivers to identify routines of persons living with ADRD, previous and current roles, habits and interests, and caregiving challenges; conducted cognitive and functional testing with persons living with ADRD to identify capacities and deficits in cognitive and physical functioning; and conducted a detailed home environment assessment. During home visits 3–8, OTs trained caregivers how to modify home environments, simplify daily activities, and communicate effectively to support capabilities; used problem-solving approaches to identify solutions for caregiver-identified caregiving challenges; and taught caregivers how to use stress reduction techniques to lower their own distress. For each targeted caregiving challenge, a written action plan or “COPE Prescription” was formulated describing treatment goals, capacities of persons living with ADRD, and specific strategies for the caregiver to implement. OT visits 9 and 10 were reserved for reviewing all that was accomplished during previous visits, and generalizing problem-solving approaches for dementia care challenges that might arise in the future (Fortinsky et al., 2016). By COPE intervention design, OT in-home visits were timed to occur approximately 12 days apart with the goal of completing all 10 OT visits within 4 months. This schedule was viewed as ideal, and it was considered more important for as many of the 10 OT visits to occur as possible, even if they could not be completed within the prescribed timeframe. A total of seven OTs, trained for this study by the originators of the COPE intervention, worked in different geographic locations in order to cover the entire Connecticut Community Care service area.

A single APN also received training for this study from the originators of the COPE intervention. In a separate home visit, timed to occur between the first and second OT in-home visit, the APN provided caregivers with information to help them identify and monitor common health-related concerns (pain detection, hydration, constipation, medication management), how to be a medical advocate, and how to plan for the future of the person living with ADRD given the trajectory of the disease. The APN also obtained blood/urine samples if possible and examined for signs of unexpressed pain and dehydration. Laboratory evaluations included complete blood count, blood chemistry, and thyroid testing of serum samples, and culture and sensitivity of urine samples. Medications were reviewed for polypharmacy and dosing appropriateness. The purpose of these clinical assessments was to rule out or identify underlying medical conditions, infections, or medication issues that may adversely affect functioning at home, as found in previous work (Gitlin et al., 2010). The APN then informed caregivers by telephone of laboratory results within 48 hr of the in-home visit and mailed two copies of the results to caregivers (one for their records and the other copy to share with physicians). For laboratory results indicative of underlying medical conditions, caregivers were asked if they preferred the APN to also fax results to the physician and/or care manager, or discuss clinical results with those care providers directly. A study geriatrician was available to consult with the APN regarding any signs, symptoms, and laboratory tests requiring further clinical interpretation before the APN provided the results to caregivers.

In order to embed COPE within the routine operation of the CHCPE, the care manager responsible for the total care plan for each CHCPE client was notified by the study coordinator when a client was assigned to the intervention group, as well as the name of the assigned OT and APN for that client. All COPE Prescriptions addressed by the OT, as well as laboratory test results stemming from APN home visits, were provided to care managers and added to clients’ health records at Connecticut Community Care. Although they played no additional role in the COPE intervention, care managers were encouraged to refer to these COPE prescriptions as part of their ongoing client care management responsibilities. Additionally, care managers and the COPE APN and OTs were encouraged to maintain communication about clients’ conditions during the time clients received the COPE intervention.

### Control Condition

Dyads randomly assigned to UC received services from the CHCPE program only. Services available in the CHCPE include emergency response systems, respite care, home-delivered meals, homemakers and companions, adult day care, nonmedical transportation, mental health counseling, care management, and assistive technology. In keeping with a pragmatic trial design, we sought to determine the value of the COPE intervention when added to customary CHCPE program services.

### Interviews With Persons Living With ADRD and Caregivers

In-person baseline (prerandomization) and 4-month postrandomization structured interviews were conducted in the home setting by trained research assistants who, by study design, were blinded to group assignment. Structured interviews contained all measures explained in the following section of the paper. By study design, these structured interviews were conducted primarily with caregivers, and with persons living with ADRD who were able to conduct their components of the structured interviews. For 12-month postrandomization structured interviews, caregivers were interviewed by telephone.

### Variable Measurements for Persons Living With ADRD and Family Caregivers

In this section, we briefly summarize outcome variables and their measurement; more details about measurement of outcome variables are found in Supplementary Table S1.

#### Primary outcome for persons living with ADRD

To determine level of *functional independence*, we used the 15-item Caregiver Assessment of Function and Upset as used in the original COPE efficacy trial (Gitlin et al., 2010).

#### Secondary outcomes for persons living with ADRD

*Activity engagement*, reported by caregivers, was measured using the five-item scale used in the COPE efficacy trial (Gitlin et al., 2010). *Behavioral and psychological symptoms*, reported by caregivers, were measured using the 14 symptom domains in the Neuropsychiatric Inventory version C (NPI-C) (Cummings, 1994; de Medeiros et al., 2010), and, to reduce respondent burden, NPI version Q (Kaufer et al., 2000) was followed by asking symptom screening questions without multiple prompts. *Quality of life*, self-reported by persons living with ADRD, was measured using the 13-item Quality of Life—Alzheimer’s Disease (QOL-AD) scale (Logsdon et al., 2002). This measure was not collected at the 12-month postrandomization interview, because that interview was conducted by telephone with the caregiver only.

#### Primary outcome for caregivers

*Perceived well-being,* the primary outcome for caregivers as in the COPE efficacy trial, was measured using the 13-item Perceived Change Index (Gitlin et al., 2006, 2010).

#### Secondary outcomes for caregivers

*Confidence in using activities* over the previous month was measured using a five-item scale developed in previous work and used in the original COPE trial (Gitlin et al., 2008, 2010). *Caregiver distress* was measured using the caregiver distress items that are part of the NPI-C, in which caregivers report the degree of distress caused by endorsed behavioral and psychological symptoms (de Medeiros et al., 2010).

#### Covariates

Prespecified covariates, chosen by balancing parsimony with evidence of their associations with proposed study outcomes, include living arrangements (live together or apart); racial and ethnic group membership (non-Hispanic white, black or African American, other group); caregiver familial relationship to person living with ADRD (spouse, adult child, other relationship); caregiver educational attainment (<high school, high school grad, >high school) as a measure of socioeconomic status; and severity of cognitive impairment of persons living with ADRD at study entry, measured using the St. Louis University Mental Status (SLUMS) examination (Tariq et al., 2006). These variables have been used previously as covariates in dementia care clinical trials including the COPE efficacy trial (Gitlin et al., 2010) and the REACH II study (Belle et al., 2006), and in a widely cited systematic review of predictors of nursing home admission among persons living with ADRD (Gaugler et al., 2009).

### Analytic Approach

Differences between the COPE and usual care groups were determined using a modified intent-to-treat analytic approach, whereby persons with ADRD and caregivers were included in analyses if they had at least one recorded outcome measure postrandomization (i.e., at 4 or 12 months). We chose a modified intent-to-treat analysis approach because there is no consensus in the literature about how best to impute missing clinical outcome values for randomized individuals who are missing outcome data. Data were analyzed primarily using analysis of covariance (ANCOVA) methods in which baseline values of outcomes were used as covariates; this approach is more efficient than analysis of change scores (Grady, 2007). The treatment group variable in the ANCOVA model tested the main hypothesis, which is whether there is an intervention versus control group mean difference in level of functional dependence at 4 months. Other outcomes at 4 months are also continuous variables and were treated the same as functional dependence level. ANCOVA models were fit in Proc GLM in SAS using OLS regression (SAS Institute, 2004). Covariates that showed no or low association with outcomes, deemed by comparing model coefficients with covariates in and out of models, were dropped for parsimony (Moher et al., 2010). To determine long-term COPE effects on outcomes, analyses based on outcome measures at 12 months postrandomization used the same ANCOVA methods as described above for 4-month outcomes, using baseline values of outcomes as covariates. We also conducted analyses to assess the extent to which any COPE effects observed at 4 months were sustained at 12 months, using linear mixed-effects models for repeated measures.

## Results

### Recruitment Results

**Figure 1.**
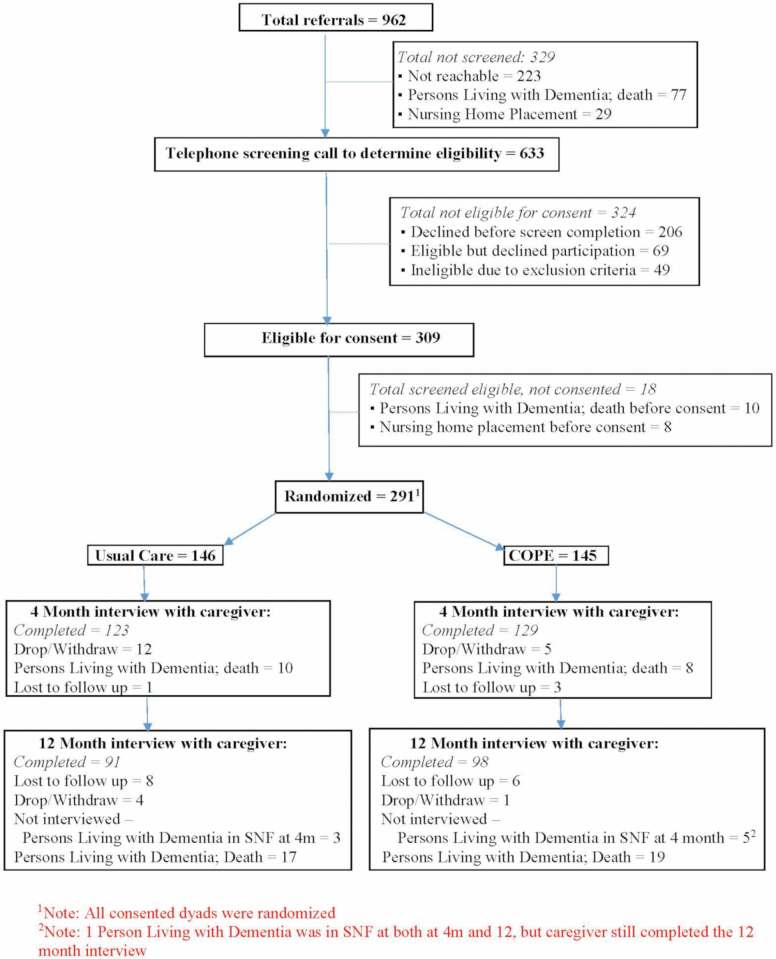
Study CONSORT chart.

Figure 1 shows the CONSORT chart for the COPE CT study. Recruitment for the study began in May 2015 and ended in February 2018. Throughout the study’s recruitment period, the research team attempted to schedule and conduct eligibility screening calls by telephone to all 962 persons living with ADRD or their caregivers who were referred by Connecticut Community Care. The research team was unable to successfully conduct eligibility screening calls for 329 referrals; of these, 223 referrals could not be reached or a screening call could not be scheduled even if contact was made, 77 persons living with ADRD died before a screening call could be initiated, and 29 persons living with ADRD were admitted to a nursing home before a screening call could be initiated.

Regarding retention of randomized dyads for study interviews at 4 and 12 months after randomization, in cases where the person living with ADRD was admitted to a nursing home prior to the next scheduled interview, the research team completed interviews with caregivers by asking them to answer questions in reference to the month prior to nursing home admission. These are considered completed interviews. In cases where the person living with ADRD died prior to the next scheduled interview, an abbreviated interview was administered to the caregiver provided they were willing to complete such an interview; these are not considered completed interviews because abbreviated interviews did not include questions measuring study outcomes. If persons living with ADRD died or were admitted to a nursing home prior to the 4-month interview, 12-month interviews were not conducted (except in the one case noted in the footnote to the CONSORT chart). One caregiver died between the baseline and the 4-month follow-up research interview and another caregiver died between the 4- and 12-month interviews. Due to the nature of the study, and questions being administered to the caregiver during the research interviews, these cases are reflected as “drops” on the CONSORT chart.

### Baseline Characteristics of Study Cohort

Table 1 presents baseline characteristics of persons living with ADRD by treatment group (total *N* = 291). Most were female (77.3%), widowed (51.5%) with a mean age of 85 years, and 16.5% were black. Most (70.5%) used Medicaid as the payment source for HCBS, representing the Medicaid waiver component of the CHCPE, while, for the remaining 30% of ADRD, state revenues paid for their HCBS. Nearly 30% has less than a high school education, and nearly 60% lived in the same household as their caregiver. Table 1 also shows that 95% of persons living with ADRD scored in the “dementia” range of the SLUMS measure, demonstrating the accuracy with which the electronic health records of Connecticut Community Care identified clients with dementia during the recruitment process. No statistically significant differences in baseline characteristics were found between persons living with ADRD randomized to the COPE group versus those randomized to the usual care group.

**Table 1.** Baseline Characteristics of Persons Living With ADRD, by Treatment Group (*N* = 291)

Characteristics	COPE group (*n* = 145)	Usual care group (*n* = 146)	*p*-value
Gender, *n* (%)			.55
Female	110 (75.9)	115 (78.8)	
Male	35 (24.1)	31 (21.2)	
Age, mean (*SD*)	85.0 (8.4)	84.9 (7.6)	.93
Race, *n* (%)			.40
Black	28 (19.3)	20 (13.7)	
White	108 (74.5)	118 (80.8)	
Other	9 (6.2)	8 (5.5)	
Marital status, *n* (%)			.87
Married	37 (25.5)	40 (27.4)	
Never married	7 (4.8)	6 (4.1)	
Widowed	75 (51.7)	75 (51.4)	
Divorced/separated	26 (17.9)	24 (16.4)	
Unknown	0	1 (0.7)	
Payment source for CHCPE services, *n* (%)			1.00
Medicaid	102 (70.3)	103 (70.6)	
State revenues (not Medicaid)	43 (29.7)	43 (29.5)	
Education level, *n* (%)			.51
Less than high school	39 (26.9)	48 (32.9)	
High school graduate	83 (57.2)	75 (51.4)	
College/postgraduate	23 (15.9)	23 (15.8)	
Functional independence			
Total score, mean (*SD*)	3.0 (1.2)	2.8 (1.1)	.38
ADL subscore, mean (*SD*)	4.1 (1.8)	4.0 (1.8)	.61
IADL subscore, mean (*SD*)	2.0 (1.0)	1.8 (0.8)	.20
Saint Louis University Mental Status (SLUMS)			
Total score, mean (*SD*)	9.0 (6.5)	9.2 (6.3)	.84
SLUMS cognitive impairment level, *n* (%)			.42
Mild cognitive impairment	9 (6.2)	6 (4.1)	
Dementia	136 (93.8)	140 (95.9)	
Self-reported quality of life, mean (*SD*)^a^			
Complete responses (*n* = 228)	2.9 (0.5)	2.8 (0.4)	.09
Missing ≤2 items (*n* = 34)	2.8 (0.5)	2.8 (0.4)	.72
Caregiver-reported 14-item Neuropsychiatric Inventory (NPI)			
Mean (*SD*)	17.8 (17.7)	18.7 (17.6)	.67
Caregiver-reported activity engagement			
Total score, mean (*SD*)	2.1 (0.46)	2.0 (0.44)	.22
Living arrangement			
Lives with caregiver	84 (57.9)	88 (60.3)	.68
Lives apart from caregiver	61 (42.1)	58 (39.7)	
Distance from caregiver in miles, mean (*SD*)	8.1 (10.4)	6.9 (9.4)	.53

*Notes*: ADL = activities of daily living; IADL = instrumental activities of daily living; ADRD = Alzheimer’s disease and related dementia; CHCPE = Connecticut Home Care Program for Elders; COPE = Care of Persons with Dementia in their Environments; SLUMS = Saint Louis University Mental Status measure.

^a^For persons living with ADRD who had two or fewer missing items on the Quality of Life—Alzheimer’s Disease (QOL-AD), missing values were replaced with mean values of all nonmissing items to calculate a score. If three or more QOL-AD items were missing, persons living with ADRD were excluded from this table and from outcome-related results shown in Tables 3 and 4, following Logsdon et al. (2002).

Table 2 displays baseline characteristics of caregivers by study group. Caregivers’ mean age was 63 years, more than one-half were daughters, followed by equal proportions of spouses and sons. Although 20% of COPE caregivers and 13.7% of usual care caregivers were black, this difference was not statistically significant. Caregivers in the COPE study group were, however, more likely to be female (76% vs 63% in the usual care group; *p* = .02). Caregivers in the usual care group showed higher levels of depressive symptoms based on Patient Health Questionnaire (PHQ-9) scores than COPE caregivers (*p* = .05). No other differences were found between COPE and usual care group caregivers (Table 2).

**Table 2.** Baseline Characteristics of Caregivers, by Treatment Group (*N* = 291)

Characteristics	COPE group (*n* = 145)	Usual care group (*n* = 146)	*p*-value
Gender, *n* (%)			.02
Female	110 (75.9)	92 (63.0)	
Male	35 (24.1)	54 (37.0)	
Age, mean (*SD*)	61.7 (11.6)	62.7 (11.0)	.46
Race, *n* (%)			.37
Black	29 (20.0)	20 (13.7)	
White	105 (72.4)	116 (79.5)	
Other	9 (6.2)	10 (6.8)	
Refused/unknown	2 (1.4)	0	
Marital status, *n* (%)			.72
Married	84 (57.9)	87 (59.6)	
Never married	27 (18.6)	23 (15.8)	
Widowed	7 (4.8)	11 (7.5)	
Divorced/separated	27 (18.6)	25 (17.1)	
Caregiver relationship to person living with ADRD, *n* (%)			.35
Spouse	25 (17.2)	27(18.5)	
Daughter	84 (57.9)	75 (51.4)	
Son	20 (13.8)	31 (21.2)	
Other	16 (11.0)	13 (8.9)	
Educational level, *n* (%)			.06
High school or less	36 (25.0)	50 (34.3)	
Some college	37 (25.7)	38 (26.0)	
College/postgraduate	71 (49.3)	58 (39.7)	
Depression (PHQ-9 score), mean (*SD*)	3.8 (4.1)	4.7 (4.3)	.05
Perceived well-being, mean (*SD*)	2.9 (0.4)	2.9 (0.4)	.60
Confidence using activities, mean (*SD*)	7.6 (2.0)	7.2 (1.9)	.11
14-item NPI Caregiver Distress score	10.3 (8.7)	10.9 (9.6)	.60

*Note*: ADRD = Alzheimer’s disease and related dementia; COPE = Care of Persons with Dementia in their Environments; PHQ = Patient Health Questionnaire; NPI = Neuropsychiatric Inventory.

### COPE Intervention Delivery Results

As Figure 1 shows, 129 dyads randomized to the COPE group completed 4-month postrandomization interviews and provided outcome data. Among these 129 dyads, we found that all except two dyads received the APN component of the COPE intervention. Additionally, 101 (78%) of these 129 dyads completed all 10 OT visits, 15 (12%) completed eight or nine visits, and 13 (10%) received seven or fewer visits. Most common reasons for not completing all OT visits were scheduling challenges that could not be resolved and caregivers determining that no additional OT visits would be helpful.

### Outcome Results at 4 Months Postrandomization

Table 3 summarizes results of analyses conducted to compare outcomes for persons living with ADRD and their caregivers at 4 months postrandomization according to whether they received the COPE intervention plus usual care (COPE) or usual care only. In these analyses, the maximum total sample size was 252 due to attrition from baseline (see CONSORT chart, Figure 1); of these, 129 were in the COPE group and 123 in the usual care group. Compared to these 252 dyads, the 39 dyads lost to attrition had the following baseline characteristics: persons with ADRD had greater levels of cognitive impairment, greater levels of physical disability, and poorer caregiver-rated quality of life; caregivers lost to attrition had lower levels of education than those retained (all *p* ≤ .05; not shown).

**Table 3.** Outcome Results at 4 Months Postrandomization, Based on General Linear Modeling^a^

Outcome results	COPE group least squares mean (95% CI) (*n* = 129)	Usual care group least squares mean (95% CI) (*n* = 123)	*p*-value
Outcomes for persons living with ADRD			
Functional independence	2.8 (2.7, 3.0)	2.8 (2.6, 2.9)	.61
Activity engagement	2.1 (2.0, 2.2)	2.1 (2.0, 2.1)	.30
Behavioral and psychological symptoms score	9.7 (5.2, 14.2)	12.7 (8.3, 17.1)	.07
Outcomes for caregivers			
Perceived well-being	3.2 (3.1, 3.3)	3.0 (2.9, 3.0)	<.001
Confidence using activities	8.2 (7.7, 8.8)	7.9 (7.4, 8.4)	.12
Distress level due to 14 behavioral and psychological symptoms of persons living with ADRD	4.7 (2.1, 7.3)	6.4 (3.9, 9.0)	.13

*Notes*: ADRD = Alzheimer’s disease and related dementia; CI = confidence interval; COPE = Care of Persons with Dementia in their Environments.

^a^Covariates for all outcome models except the model for self-reported quality of life of persons living with ADRD included baseline values of: corresponding outcome; caregiver gender, race, ethnicity, education, relationship to person living with ADRD, depressive symptoms, living arrangement, baseline hours/week supervising person living with ADRD, and Saint Louis University Mental Status (SLUMS) score of person living with ADRD. Covariates for the self-reported quality of life of persons living with ADRD model: baseline value of self-reported quality of life; person living with ADRD age, gender, race, ethnicity, education, relationship to caregiver, living arrangement, and SLUMS score. For all outcomes, initial models included all covariates simultaneously, and final models included statistically significant covariates in initial models.

Table 3 shows that, among outcomes for persons living with ADRD, there were no statistically significant COPE effects after 4 months; however, there was a strong trend toward lower scores on the measure of behavioral and psychological symptom frequency and severity for those receiving COPE compared to the usual care group at four months (adjusted means = 9.7 vs 12.7, respectively; *p* = .07). Among caregivers, those receiving COPE reported statistically significantly greater perceived change for the better than usual care counterparts (3.2 vs 3.0, respectively; *p* < .001).

### Outcome Results at 12 Months Postrandomization

Table 4 summarizes results of analyses conducted to compare outcomes by treatment group at 12 months postrandomization. Among persons living with ADRD, level of activity engagement in the COPE group was statistically significantly higher than in the usual care group (*p* = .02). No other outcomes were statistically significantly different by treatment group. We also found, based on results from all mixed effects models, that no COPE effects observed at 4 months postrandomization were sustained at 12 months postrandomization (analyses not shown).

**Table 4.** Outcome Results at 12 Months Postrandomization Based on General Linear Modeling^a^

Outcome results	COPE group least squares mean (95% CI) (*n* = 96)^b^	Usual care group least squares mean (95%CI) (*n* = 89)^b^	*p*-value
Outcomes for persons living with ADRD			
Functional independence	2.6 (2.5, 2.8)	2.5 (2.4, 2.7)	.18
Activity engagement	2.1 (2.0, 2.1)	1.9 (1.9, 2.0)	.02
Behavioral and psychological symptoms score	15.8 (12.8, 18.7)	18.8 (15.8, 21.9)	.16
Self-reported quality of life	2.3 (2.2, 2.4)	2.3 (2.2, 2.4)	.42
Outcomes for caregivers			
Perceived well-being	3.0 (2.9, 3.1)	2.9 (2.8, 3.0)	.31
Confidence using activities	7.7 (7.3, 8.1)	7.5 (7.0, 7.9)	.23
Distress level due to behavioral and psychological symptoms of person living with ADRD	8.2 (6.2, 10.1)	9.0 (7.0, 11.0)	.42

*Notes*: ADRD = Alzheimer’s disease and related dementia; CI = confidence interval; COPE = Care of Persons with Dementia in their Environments.

^a^See Table 3 footnote for details about covariates for all outcome models. ^b^Sample sizes in analyses shown in this table differ from figures shown in the CONSORT chart (Figure 1) because two caregivers in each treatment group who participated in a 12-month postrandomization interview (shown as 98 and 91 in COPE and Usual Care groups, respectively, in Figure 1) provided incomplete data available for analyses.

## Discussion and Implications

COPE was previously tested and shown to be efficacious on a number of outcomes related to persons living with ADRD and their caregivers. This is the first study to test COPE as an embedded program in a service delivery system, specifically a Medicaid- and state-revenue funded HCBS program, and to evaluate its effectiveness using the rigor of randomized trial methodology. Study participants included persons living with ADRD and their informal caregivers who received services from this HCBS program. We found that caregivers who received HCBS plus COPE, compared to those who received HCBS alone, improved significantly from baseline to 4 months postbaseline in their perceived well-being, specifically in their reported ability to manage day-to-day care challenges, their affective stance, and somatic well-being. We also found that persons living with ADRD who received HCBS plus COPE experienced decreased frequency and severity of behavioral and psychological symptoms after 4 months compared to those not receiving COPE, at a borderline level of statistical significance. Finally, we found that after 12 months, persons living with ADRD who received HCBS plus COPE had a significantly higher level of engagement in meaningful activities than those not receiving COPE.

These findings suggest that COPE has added value for persons living with ADRD and their caregivers who already receive Medicaid- and state-revenue funded HCBS, such as care management, homemaker, personal care assistance, and adult day services. COPE demonstrated a positive effect on caregivers’ well-being related to managing dementia-related symptoms, the primary outcome for caregivers in this study, as it was found in the original COPE efficacy trial (Gitlin et al., 2010). This effect is consistent with COPE principles underlying the strategies provided by the nurse and OT to caregivers, which are intended to support their overall well-being (Fortinsky et al., 2016; Gitlin et al., 2010). For persons living with ADRD, the value lies in improved ability to engage in meaningful activities and reduced levels of behavioral and psychological symptoms.

The lack of impact of the COPE intervention on functional independence, the primary outcome for persons living with ADRD which was found to improve among COPE recipients in the original COPE efficacy trial (Gitlin et al., 2010), deserves further comment. No treatment group differences were found in the total functional independence score, or in the activities of daily living or instrumental activities of daily living subscores. We found in this study that those receiving COPE and those in the HCBS-only group experienced similar levels of functional decline from study baseline to both 4 and 12 months postbaseline. Both treatment groups started this study with low baseline levels of functional independence, suggesting a floor effect for functionality and reflecting the study’s target population of older adults with dementia already receiving HCBS because they were deemed at-risk for long-term nursing home admission. In contrast, participants in the original COPE efficacy trial were community-dwelling volunteers with cognitive impairment but were not deemed nursing home eligible (Gitlin et al., 2010). For lower-functioning older adults living with dementia and enrolled in Medicaid- and/or other publicly funded HCBS programs, study results suggest that there is merit in considering how goals of COPE might be modified to optimize functional independence.

Study findings have implications for efforts to expand the availability of the COPE intervention throughout Medicaid HCBS waiver programs for older adults at high risk for long-term nursing home admission. More than 40 states operate Medicaid HCBS waiver programs for older adults at risk for long-term nursing home admission (Watts et al., 2020) and, although national figures are unavailable, based on the 25%–30% figure in Connecticut, it is likely that sizeable proportions of this client population in other states live with ADRD, with family caregivers interacting with the service system to help keep them at home. Lessons learned about implementation of COPE in this study could be applied to the context of Medicaid HCBS programs in other states that might wish to address the needs of this target population with an efficacious and effective dementia care program.

These implications for widespread adoption of COPE are highly relevant to the nascent field of pragmatic trials in the field of dementia care for community-dwelling older adults and their family caregivers. Research on dementia care is at an important inflection point where efficacious nonpharmacologic interventions must be made available to researchers and stakeholders in health care systems (Gitlin, Baier, et al., 2020), as well as in systems that provide long-term services and supports. In the United States, pragmatic trial collaborations between investigators and dementia care stakeholders are poised to increase considerably in the next few years, due to recent initiatives funded by the National Institute on Aging (NIA), including the Pragmatic Alzheimer’s Disease and AD-Related Dementias Clinical Trials Collaboratory (NIA IMPACT Collaboratory) and Roybal Centers focused on family caregivers to older adults living with chronic disease and disability, including dementia.

We also emphasize successes in carrying out this study via the use of pragmatic trial elements when working in partnership with a large care management organization responsible for coordinating HCBS services for the target population. Study eligibility criteria for persons living with ADRD accurately reflected characteristics of clients served by the care management organization and were successfully identified at the client level using the electronic health records of the care management organization. Moreover, care managers were able to offer study participation to their clients and caregivers as part of their usual workflow, and COPE prescriptions developed by OTs as well as results of clinical assessments and laboratory results obtained by the COPE nurse were successfully transferred to the care management records of persons living with ADRD. These findings demonstrate that the practical logistics of embedding a dementia care intervention like COPE can be successfully designed to enable a health care system or social service organization with electronic health records to offer COPE to persons living with ADRD and caregivers in a pragmatic fashion. Further work is needed to incorporate additional pragmatic trial elements into efforts to fully embed COPE into service settings, such as defining and measuring outcomes that can be easily tracked within electronic health records of Medicaid and other publicly funded HCBS programs.

This study had limitations that are relevant to future efforts to embed interventions such as COPE into health care systems and service settings such as Medicaid HCBS programs. First, this study included only English-speaking persons living with dementia and caregivers because at this time COPE is available only in English. Care managers from Connecticut Community Care urged the study team to develop a Spanish language version of the COPE intervention because of the growing number of Spanish-speaking clients and family members in their caseloads. Second, due to the practical realities of scheduling OT visits with caregivers of persons living with ADRD who were also receiving multiple in-home and community-based services from the Medicaid- and state revenue-funded HCBS program, it took longer than 4 months to complete all OT visits for the majority of dyads in the COPE group. This experience suggests that in pragmatic trials, the length of the OT component of the COPE intervention should be expected from the outset to have a longer duration than the 4 months as originally designed, and that evaluative strategies should be modified accordingly. Third, we were unable to discern specific type of dementia diagnosis, which remains an important challenge for future pragmatic trials when existing data might not contain dementia diagnostic codes. Finally, outcomes specific to persons living with ADRD and their caregivers used in this effectiveness study were chosen intentionally to closely mirror those used in the original COPE efficacy trial; however, future efforts to embed COPE in service systems should strongly consider using additional outcomes that are deemed relevant to persons living with ADRD, caregivers, and service system stakeholders in a constantly changing dementia care environment.

In conclusion, in this study, we demonstrated that, through carefully designed partnerships between investigators and service providers, an efficacious dementia care intervention can be embedded and offered in a pragmatic fashion within a publicly funded HCBS program, with measurable benefit particularly to family caregivers of community-dwelling older adults living with dementia. Further research is necessary to understand cost and financial impact of COPE in this service environment; acceptability of the program by care managers, staff, and administrators; whether certain subgroups are more likely to benefit from receiving COPE; and strategies for scaling up and disseminating COPE in Medicaid- and other publicly funded HCBS programs throughout the country.

## Supplementary Material

igaa053_suppl_Supplementary_Materials
