# Supplementary material for: Effectiveness of the Care of Persons With Dementia in Their Environments Intervention When Embedded in a Publicly Funded Home- and Community-Based Service Program
Source: Innov Aging. 2020 Oct 26;4(6):igaa053. doi: 10.1093/geroni/igaa053 (PMC7745768; doi:10.1093/geroni/igaa053)
Supplement: igaa053_suppl_Supplementary_Materials [file igaa053_suppl_supplementary_materials.docx]

Online Supplementary Material for Accepted Publication in *Innovation in Aging*:

**Effectiveness of the Care of Persons with Dementia in their Environments (COPE) Intervention When Embedded in a Publicly-Funded Home and Community-Based Service Program**

Richard H. Fortinsky, PhD^1,*^, Laura N. Gitlin, PhD, FGSA, FAAN^2^, Laura T. Pizzi, PharmD, MPH^3^, Catherine Verrier Piersol, PhD, OTR/L^4^, James Grady, DrPH^5^, Julie T. Robison, PhD^1^, Sheila Molony, PhD, APRN^6^, Dorothy Wakefield, MS, PStat^1^

1. Center on Aging, School of Medicine, University of Connecticut, Farmington, Connecticut

2. College of Nursing and Health Professions, Drexel University, Philadelphia, Pennsylvania

3. Center for Health Outcomes Research, Rutgers University, Piscataway, New Jersey

4. College of Health Professions, Thomas Jefferson University, Philadelphia, Pennsylvania

5. Department of Public Health Sciences, School of Medicine, University of Connecticut, Farmington, Connecticut

6. School of Nursing, Quinnipiac University, North Haven, Connecticut

*Address correspondence to: Richard H. Fortinsky, PhD, UConn Center on Aging, UConn Health, 263 Farmington Ave., Farmington, CT 06030-5215; email: [Fortinsky@uchc.edu](mailto:Fortinsky@uchc.edu)

**Table S1. Outcome Variable Measurement Details**

| **Variable** | **Measure** | **Measurement notes** | **Sources** |
| --- | --- | --- | --- |
| **Persons with ADRD** |  |  |  |
| Functional independence | Caregiver Assessment of Function and Upset (CAFU) | Caregiver-reported measure  Total score=15 items  Items include 7 self-care activities of daily living (ADLs): bathing, dressing upper/lower body, using the toilet, grooming, eating, and getting into/out of bed; and 8 instrumental ADLs (IADLs); telephone use, shopping, meal preparation, housework, laundry, transportation, taking medicine, and managing finances.  ADL subscore=7 items  IADL subscore=8 items  Item response range=1(needs complete help) to 7 (completely independent)  Total summed scores divided by number of items | Gitlin et al, 2010 |
| Activity engagement | Activity engagement scale | Caregiver-reported measure  5 items  Item response range=1 (never) to 3 (often)  Total summed score divided by number of items | Gitlin et al, 2010 |
| Quality of life | Quality of Life-Alzheimer’s Disease (QOL-AD) | Self-reported measure  13 items  Item response range=1 (poor) to 4 (excellent)  Total summed score divided by number of items  Collected only at baseline and 4-month post-randomization in-person interviews | Logsdon et al, 2002 |
| Behavioral and psychological symptoms | Neuropsychiatric Inventory Version C (NPI-C) for item content | Caregiver-reported measure  14 items/symptoms  For each endorsed symptom, frequency response range=0 (never) to 4 (at least daily); Severity response range=0 (no severity) to 3 (marked severity)  Score calculated by multiplying frequency times severity for each item and summed across all items. | Cummings et al, 1994;  de Medeiros et al, 2010; Kaufer et al, 2000 |
| **Caregivers** |  |  |  |
| Perceived well-being | Perceived change index | Caregiver self-reported  13 items  Caregivers rate change in ability to manage dementia, emotional status (anger, distress), and somatic symptoms (energy, sleep quality) in the past month using 5-point scales, with responses ranging from 1 (got much worse) to 5 (improved a lot). Total mean score derived by summing across items and dividing by the number of items. Higher scores indicate greater improvement. | Gitlin et al, 2006;  Gitlin et al, 2010 |
| Confidence in using activities | Activity confidence index | Caregiver self-reported  5 items  Caregivers rate each item (e.g., involve persons living with ADRD in meaningful activities; use activities to distract persons living with ADRD) from 0 (not confident) to 10 (very confident)  Total mean score derived by summing across items and dividing by number of items. | Gitlin et al, 2008  Gitlin et al, 2010 |
| Distress due to behavioral and psychological symptoms | Caregiver distress index | Caregiver self-reported  14 items  For each NPI-C symptom endorsed, caregiver rates degree of distress caused by that symptom, ranging from  0 (not distressing) to 4 (extremely distressing)  Total score derived by summing across items. | de Medeiros et al, 2010 |
